# Supplementary material for: Transcriptome Analysis of Developmental Gene Expression in Thesium chinense Turcz
Source: Plants (Basel). 2025 Aug 16;14(16):2549. doi: 10.3390/plants14162549 (PMC12389193; doi:10.3390/plants14162549)
Supplement: Supplementary file 1 [file plants-14-02549-s001.zip › Supplementary Figures and Table Legends.pdf]

Supplementary Figures

Figure S1: Growth phenotypes of *T. chinense* in one and two years. The left panel shows the phenotypes of the whole plant at the first year of life, and the right panel shows the phenotypes of the whole plant at the second year of life.

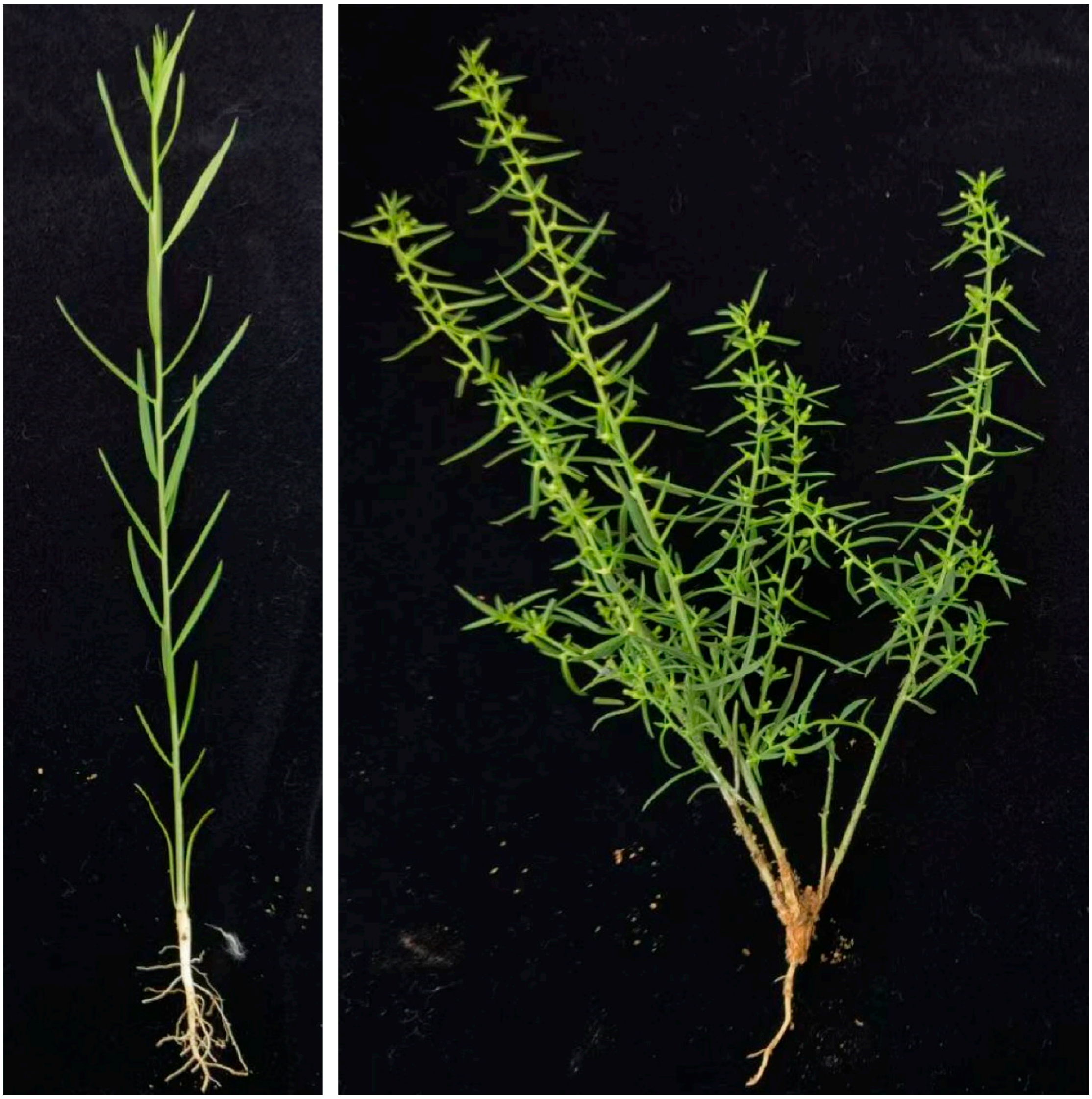

Figure S2: Volcano plot of Tc1_S vs Tc1_F, Tc1_F vs Tc1_Fr, Tc1_S vs Tc1_Fr, Tc2_S vs Tc2_F, Tc2_F vs Tc2_Fr, Tc2_S vs Tc2_Fr, Tc1_S vs Tc2_S, Tc1_F vs Tc2_F and Tc1_Fr vs Tc2_Fr.

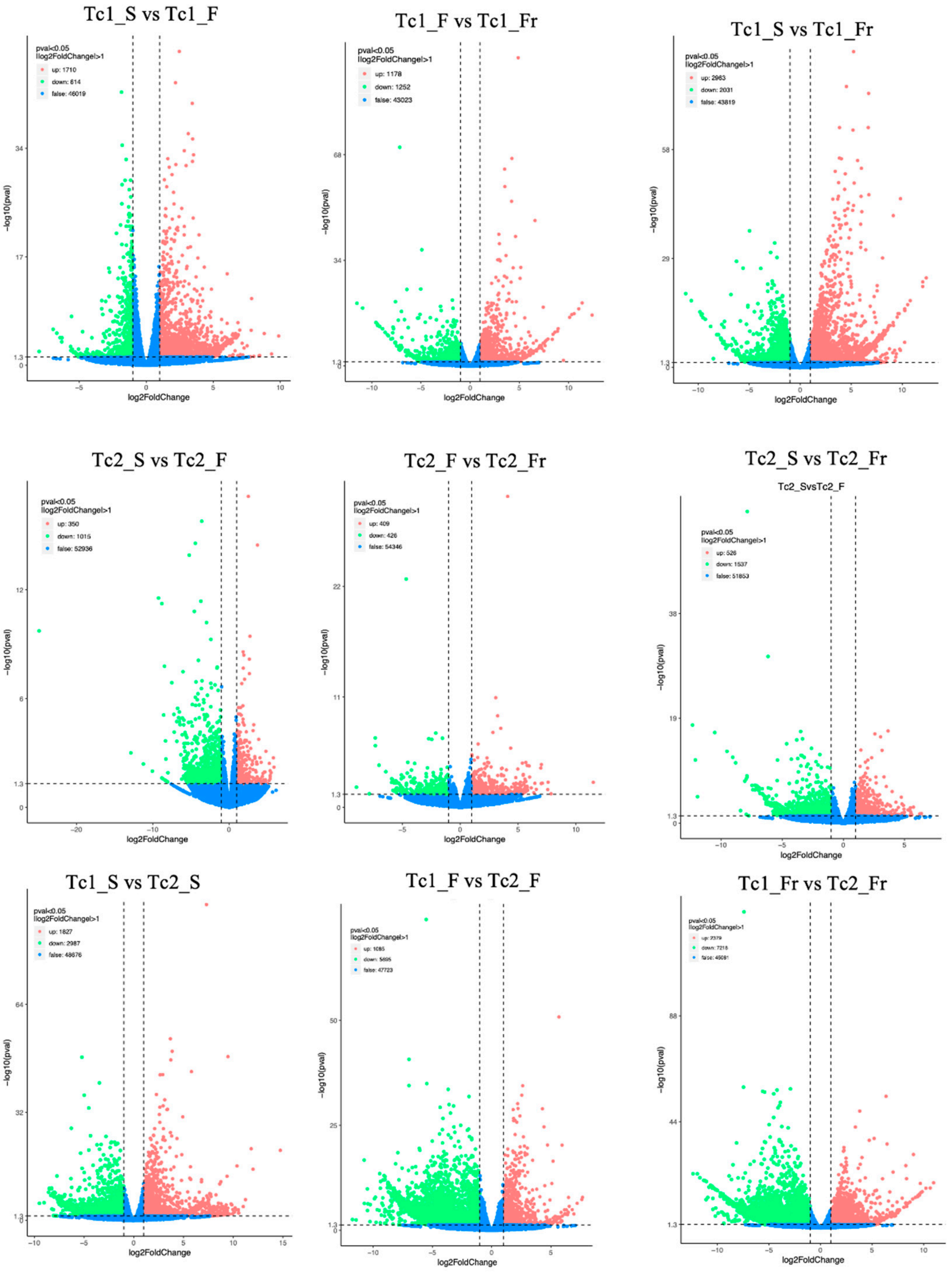

Figure S3: All transcription factors exhibit expression trends over time. Different samples are shown on the X-axis and relative expression is shown on the Y-axis. Broken lines were merged and described as fitted curves.

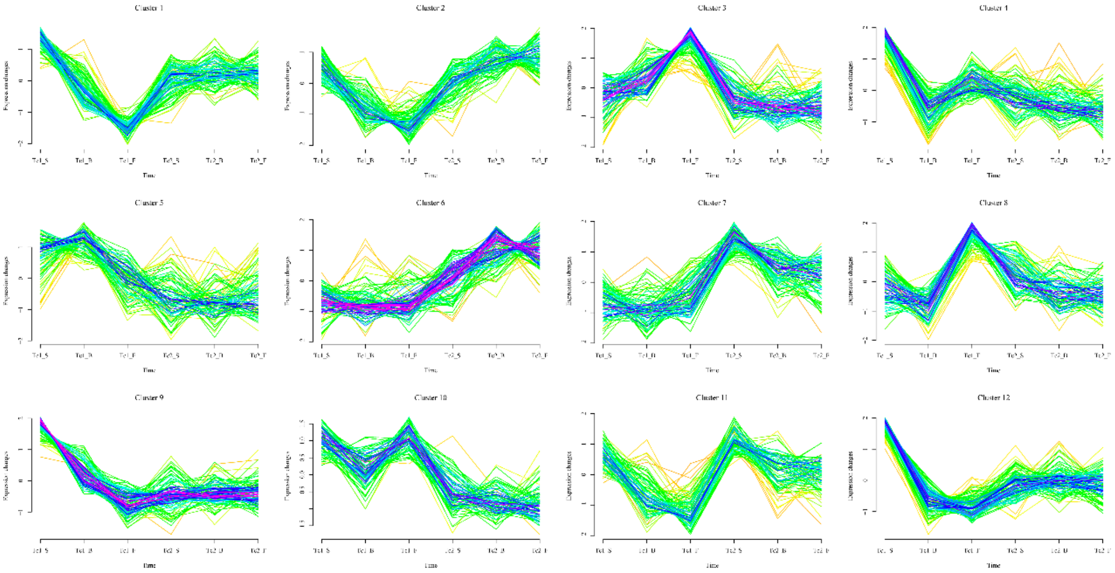

Figure S4: Co-expression Network Modules gene classification. A. Selection of soft threshold in co-expression network construction. **B**. Module division.

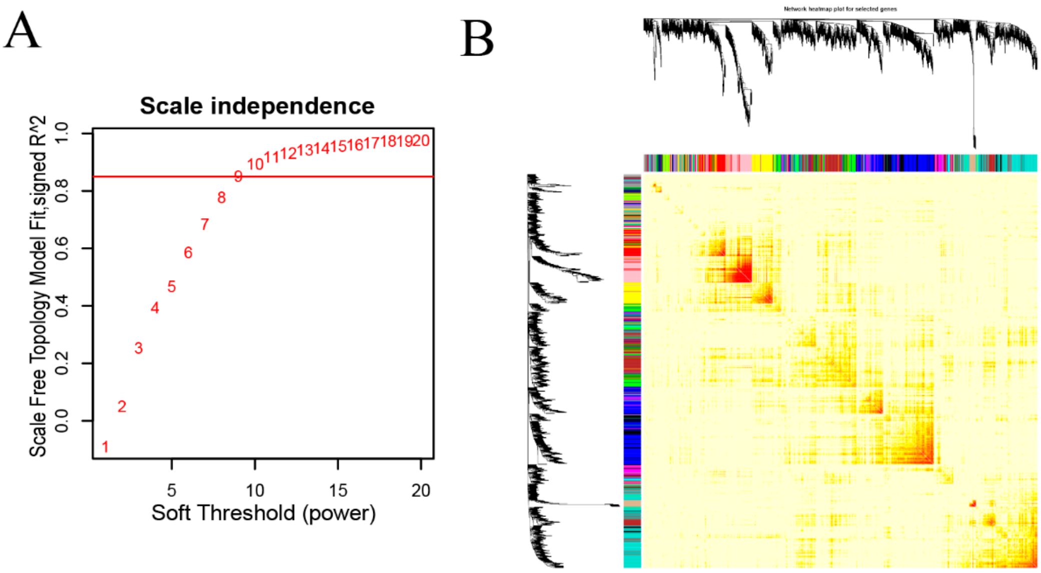

Figure S5: KEGG functional annotation of flavonoid biosynthesis. The expression levels of genes related to flavonoid biosynthesis are mapped to their corresponding genes. Image pathways were derived from KEGG metabolic pathway database, squares represent genes and circles represent intermediate metabolites.

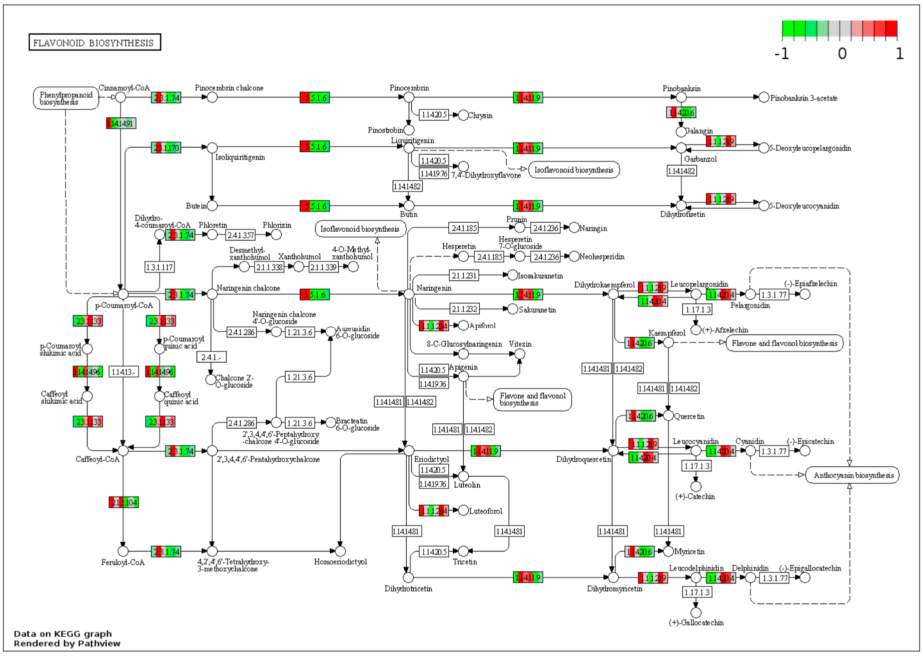

Figure S6: Expression trends of differentially expressed genes over time. Different samples are shown on the X-axis and relative expression is shown on the Y-axis. Broken lines are described as fitted curves, and the redder the color, the closer to the fitted curve.

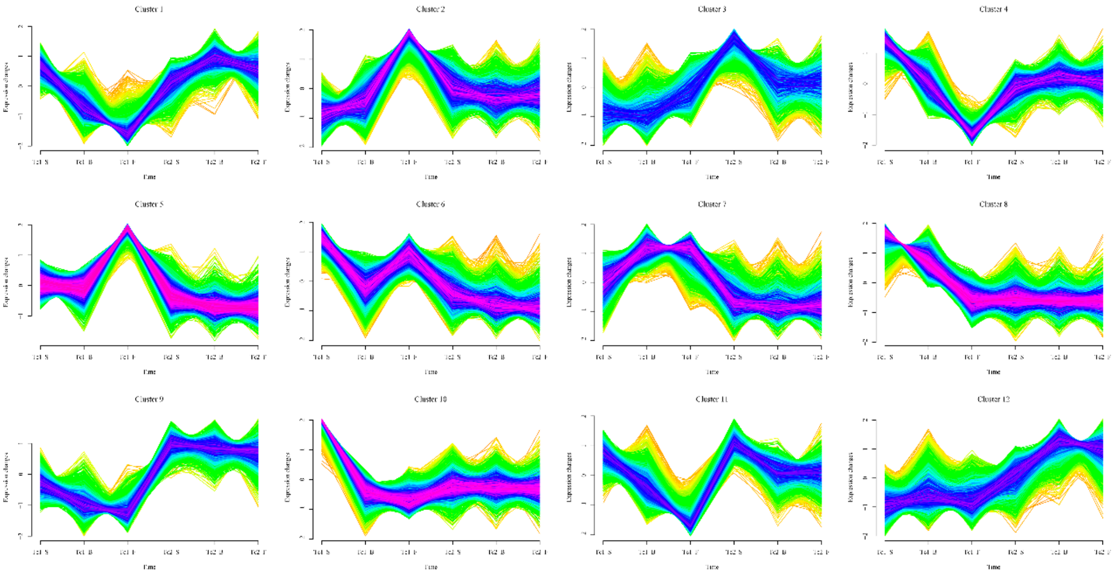

Figure S7: ***FAR1*** **regulates the co-expression network of the** ***CYP98A3*** **gene.**

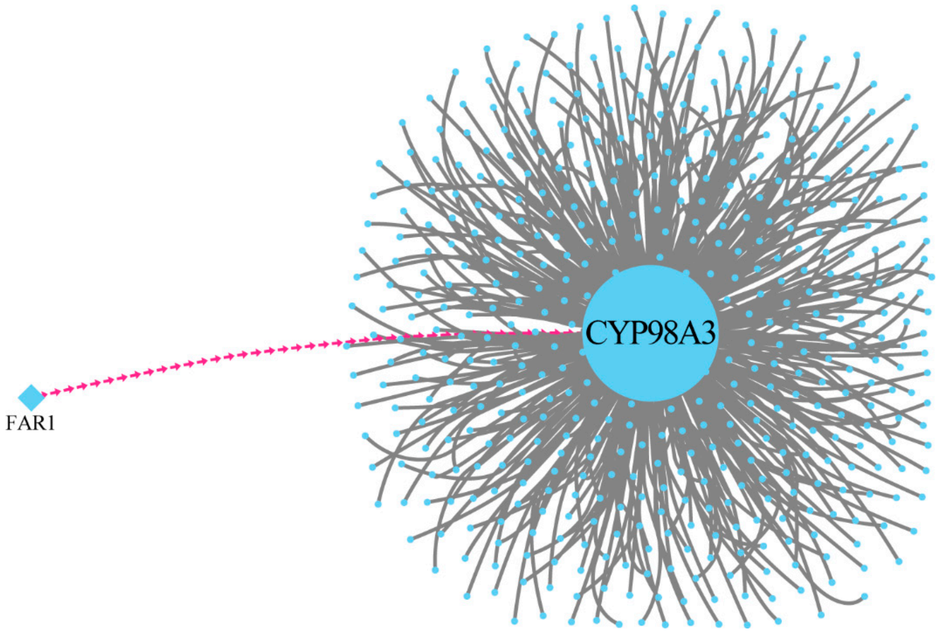

Supplementary Table Legends

Table S1: Transcriptome assembly related information for transcript and unigene.

Table S2: Annotation information for the unigenes from the transcriptome.

Table S3: Prediction of CDS information for unigenes. This appendix describes the length of unigenes, the homologous matching method, the matched gene id and CDS length and the protein length, respectively**.**

Table S4: TFs annotation information. This appendix describe the gene functions annotated by transcription factors in different databases.

Table S5: TFs regulatory network.

Table S6: Primer sequences for all relevant genes.
